# Case Report: short stature, kidney anomalies, and cerebral aneurysms in a novel homozygous mutation in the *PCNT* gene associated with microcephalic osteodysplastic primordial dwarfism type II

**DOI:** 10.3389/fendo.2023.1018441

**Published:** 2023-05-10

**Authors:** Maddalena Petraroli, Antonio Percesepe, Maria Piane, Francesca Ormitti, Eleonora Castellone, Margherita Gnocchi, Giulia Messina, Luca Bernardi, Viviana Dora Patianna, Susanna Maria Roberta Esposito, Maria Elisabeth Street

**Affiliations:** ^1^ Unit of Paediatrics, Department of Medicine and Surgery, University and University Hospital of Parma, Parma, Italy; ^2^ Medical Genetics Unit, University of Parma, Parma, Italy; ^3^ Department of Clinical and Molecular Medicine, “Sapienza” University of Rome, Rome, Italy; ^4^ Unit of Radiology, University Hospital of Parma, Parma, Italy

**Keywords:** growth, microcephalic osteodysplastic primordial dwarfism, cerebral aneurysms, bone dysplasia, short stature, PCNT gene, MOPDII, intellectual disability

## Abstract

We report the case of a boy (aged 3 years and 7 months) with severe growth failure (length: -9.53 SDS; weight: -9.36 SDS), microcephaly, intellectual disability, distinctive craniofacial features, multiple skeletal anomalies, micropenis, cryptorchidism, generalized hypotonia, and tendon retraction. Abdominal US showed bilateral increased echogenicity of the kidneys, with poor corticomedullary differentiation, and a slightly enlarged liver with diffuse irregular echotexture. Initial MRI of the brain, performed at presentation, showed areas of gliosis with encephalomalacia and diffused hypo/delayed myelination, and a thinned appearance of the middle and anterior cerebral arteries. Genetic analysis evidenced a novel homozygous pathogenic variant of the pericentrin (PCNT) gene. PCNT is a structural protein expressed in the centrosome that plays a role in anchoring of protein complexes, regulation of the mitotic cycle, and cell proliferation. Loss-of-function variants of this gene are responsible for microcephalic osteodysplastic primordial dwarfism type II (MOPDII), a rare inherited autosomal recessive disorder. The boy died at 8 years of age as a result of an intracranial hemorrhage due to a cerebral aneurism associated with the Moyamoya malformation. In confirmation of previously published results, intracranial anomalies and kidney findings were evidenced very early in life. For this reason, we suggest including MRI of the brain with angiography as soon as possible after diagnosis in follow-up of MODPII, in order to identify and prevent complications related to vascular anomalies and multiorgan failure.

## Background

The most common form of microcephalic primordial dwarfism **(**MPD) is MOPDII, which has several distinctive clinical features compared to the other forms, such as more severe growth impairment with skeletal dysplasia, and global vascular and metabolic disease with hypertension and insulin resistance. Microcephaly, a high forehead with receding hairline, a prominent beaked nose with ocular proptosis, micrognathia with relatively proportioned small mouth, dental dysplasia, and high squeaky voice represent the main phenotypic facial features (1, 2). Radiological abnormalities are also typically observed in MOPDII patients, with a tendency to worsen over time: at birth, children present with a high, narrow pelvis, small iliac bones, and flat acetabulum with subsequent femoral head subluxation or dislocation. As the child grows, skeletal changes become more severe, with possible proximal femoral epiphysiolysis, *coxa vara*, flared appearance of the metaphysis of distal long bones, progressive disharmony of the short stature due to mesomelic shortening of the limbs, and general bone age retardation as a result of delayed ossification (3, 4). Moreover, MOPDII is associated with increased vascular risk due to cerebral vessel anomalies that become responsible for early mortality (5, 6).

To date, at least 13 different genes responsible for specific disorders belonging to the MPD group have been identified. All these genes play a fundamental role in regulating centrosome activity, genome replication, and DNA damage response, with a strong overlap in function in the proteins encoded. Nevertheless, each disorder belonging to this group of skeletal dysplasias shows distinctive features depending on the gene involved (7). In addition to MOPDII and Seckel syndrome, MPD includes MOPD types I/III and Meier-Gorlin syndrome; despite distinctive molecular bases, these conditions share key clinical characteristics, such as extreme global growth impairment with severe short stature, microcephaly, and intellectual disability.

MOPDII **(**OMIM #210720) is a rare inherited autosomal recessive disorder caused by homozygous or compound heterozygous mutations in the pericentrin (*PCNT*) gene on chromosome 21q22 (8, 9).

**Figure 1:**
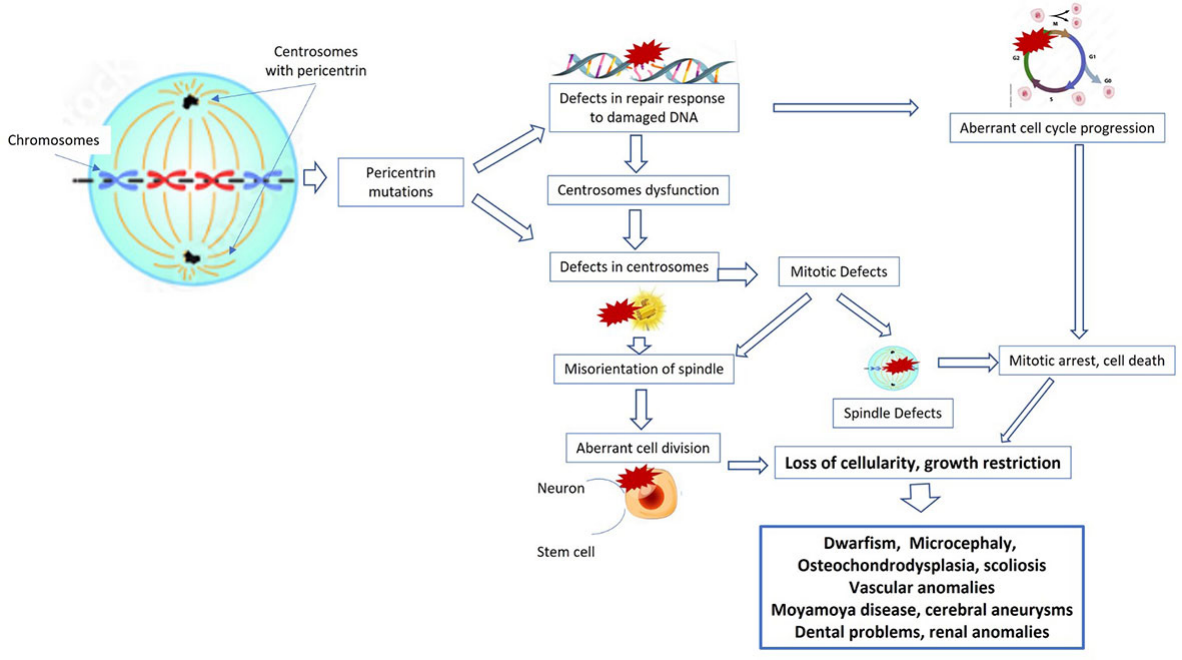
Cellular pathways and mechanisms implicated in primordial dwarfism. PCNT and other proteins are required for normal functioning of the centrosome, and therefore mutations in these genes also impair centrosome function. Moreover, pericentrin mutations can cause defects in the repair response to damaged DNA, with consequent aberrant cell cycle progression, mitotic arrest, and cell death. All these mechanisms lead to loss of cellularity, to growth restriction, and then to dwarfism, microcephalia, and bone, renal, and vascular anomalies.

*PCNT* mutations have previously been found to be associated with both Seckel syndrome and MOPDII, although the most recent analyses on larger case series have confirmed the specificity of pericentrin involvement in patients affected by MOPDII (10) (**Figure 1**). So far, over 150 individuals have been diagnosed with MOPDII (1), and 1147 variants of the *PCNT* gene have been described at present: 670 of these are classified as variants of uncertain clinical significance (VUS), 360 as benign, 90 as pathogenic, and 27 as probably pathogenic (11).

Here, we describe the case of a Moldovan child who moved to Italy at the age of three years, and who was diagnosed with MOPDII caused by a novel homozygous mutation in the *PCNT* gene (c.3019_3020del, p.Leu1007Serfs*50), which is responsible for an early termination site in protein synthesis; thus, premature truncation of protein synthesis was predicted, with a subsequent severe phenotype to be aware of.

## Case report

**Figure 2:**
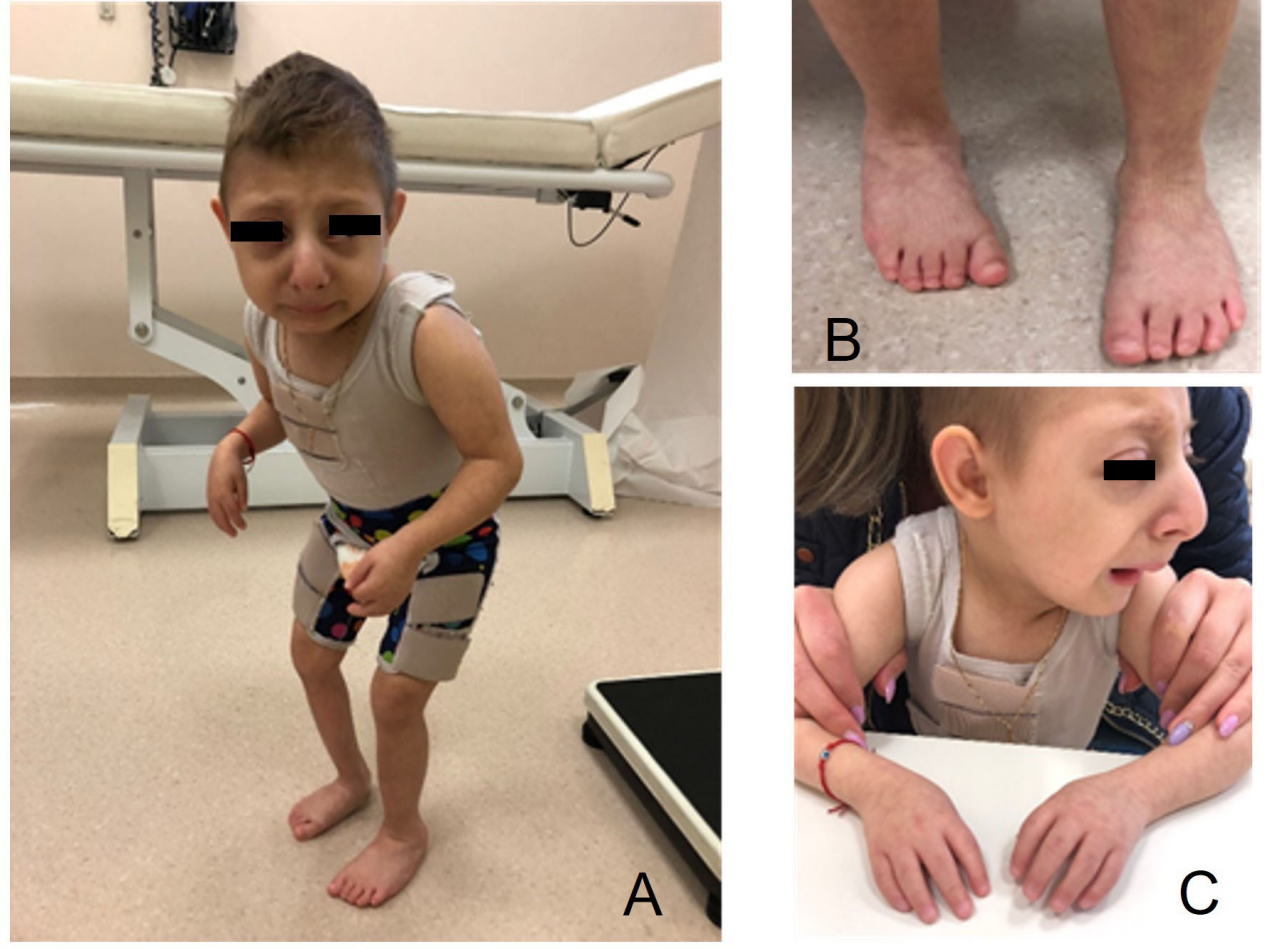
Features of the child with microcephalic osteodysplastic primordial dwarfism type II (MOPDII) at age 7 years. **(A)** Extreme short stature, arthrogryposis of the hands, and the forced posture can be observed; **(B)** equinus right foot; **(C)** high forehead with ocular proptosis, prominent beaked nose, and micrognathia.

The child was referred to our pediatric endocrinology outpatient clinic because of severe short stature and absence of catch-up growth since birth. All clinical features of MOPDII and of our patient are summarized in **Table 1**. He presented with intellectual disability and distinctive craniofacial features. At our first evaluation (when the patient was 3 years and 7 months old), his length was 62.4 cm (-9.53 SDS by WHO references), he weighed 5.15 kg (-9.36 SDS by WHO references), and he presented with microcephaly (43 cm, -4.8 SDS by WHO references). He had a high forehead with ocular proptosis, a prominent beaked nose, micrognathia, a relatively proportioned small mouth with multiple dental caries, brachymesophalangy, arthrogryposis of the hands, and equinus right foot. The boy also had a micropenis, hypotrophic-like right testis, and non-palpable left testis. On ultrasound the left testis was found to be intra-abdominal, with regular morphology and echotexture, while the right testis was observed within the inguinal canal, confirming clinical cryptorchidism. He also presented with generalized hypotonia and resistance to dorsiflexion of the right lower limb due to the presence of tendon retraction. Brisk and asymmetrical patellar osteotendinous reflexes were observed (**Figure 2**).

**Table 1:** Features of Microcephalic Osteodysplastic Primordial Dwarfism Type II compared with the patient described.

Feature		Our patient
Extreme pre- & postnatal growth restriction: IUGR, severe short stature		**Present**
Microcephaly		**Present**
Skeletal dysplasia: hip deformity &/or scoliosis in addition to osteochondrodysplasia. Dysplasia may be difficult to recognize in newborn period.		**Present, with brachy-mesophalangy, arthrogryposis of the hands and equinus right foot**
Small, loosely rooted teeth: typically secondary teeth are more affected than primary teeth.		**Present, with multiple caries**
Hematologic	Anemia, Thrombocytosis	Absent
Cerebrovascular	Aneurysms: lifelong risk, median age 9.3 years	**Present**
	Moyamoya vasculopathy: mainly in younger ages	**Present**
Cardiovascular	Hypertension: Median age 13 yrs	Absent
	Hypercholesterolemia: Median age 18 yrs	Absent
	Cardiac malformations ASD, VSD, PFO	Absent
	Coronary artery disease w/premature Mis: Median age of MI 24 yrs	Absent
Renal	Chronic kidney disease Renal transplantation documented in 2 persons	Absent
	Accessory renal arteries: only described in males	Absent
	Renal vascular disease: Renal artery stenosis, aneurysm	Absent
	AR PKD-like features (hyperechoic thickening of adipose tissue of the perirenal space)	**In addition**
Liver	Liver enlargement, diffuse irregular echostructure	**In addition**
	Cryptorchidism / retractile testes	**Present**
Genital	Hypospadias	Absent
	Micropenis	**In addition**
Endocrine	Insulin resistance &/or diabetes mellitus: median age 11 yrs	Absent
Muscolo-skeletal	generalized hypotonia; tendon retraction	**In addition**
	Borderline/low-normal intellectual function: More impairment in those who have had strokes	Absent
Cognitive ability	ADHD Not yet definitively evaluated in large studies	Absent
	Intellectual disability	**In addition**

Modified from Duker et al (1) ADHD, attention-deficit/hyperactivity disorder; AR-PKD, Autosomal recessive polycystic kidney disease, ASD, atrial septal defect; IUGR, intraterine growth restriction; MI, myocardial infarction; PFO, patent foramen ovale; VSD, ventricular septal defect.

Medical history evidenced a pregnancy characterized by severe intrauterine growth retardation (IUGR). The mother reported that he was born around 28 weeks of gestational age, and that she discovered pregnancy late. Weight at birth was 890 **g** (he was born in Moldova, then moved to Italy). He was admitted for congenital CMV infection, but few data were available from the records brought by the mother. Karyotype was 46,XY. He was breastfed during the first weeks of age, and subsequently received formula milk. He was admitted to hospital again at 18 months of age for pneumonia.

At the first evaluation at our clinic, the patient was 3 years and 7 months old. He would eat only smooth foods and exhibited slow and difficult chewing. After dental eruption, fragmentation of the teeth was observed.

**Figure 3:**
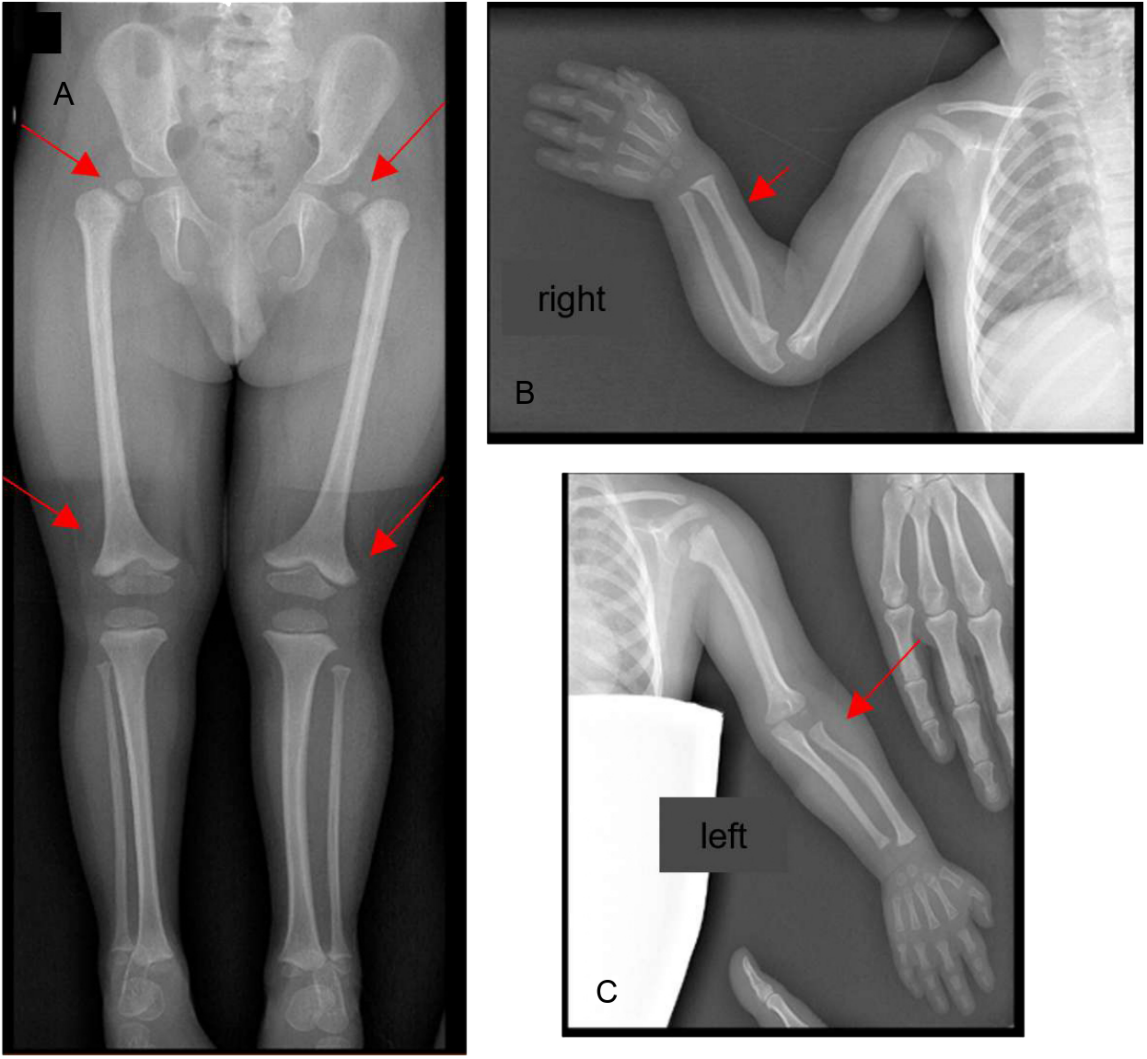
Bone Rx -ray anomalies at 3 years of age: **(A)** pelvis and lower limbs: bilateral subluxation of the femoral heads, more pronounced on the left with wide acetabular angles; flared appearance of the distal metaphysis of both femurs; **(B, C)** shoulders and upper limbs: dislocation of the humeral ossification center, convex shape of the radius bilaterally. The red arrows point at the anomalies described.

Considering the severe growth impairment, a Seckel’s syndrome spectrum disorder was initially suspected, and the child underwent biochemical and radiological investigations. A total-body skeletal survey showed multiple and distinctive bone anomalies, including bone age delay (according to the Greulich and Pyle standards, this was 1 year and 3 months at a chronological age of 3 years and 10 months); dislocation of the humeral ossification center; convex shape of the radius bilaterally; and bilateral subluxation of the femoral heads, more pronounced on the left (which evolved to luxation within three years), with wide acetabular angles and flared appearance of the distal metaphysis of both femurs as a consequence of severe skeletal dysplasia (**Figure 3**).

Abdominal ultrasound at 3 years of age showed normal echostructure and size of both kidneys, but at 6 years of age, bilateral increased echogenicity was present, with poor corticomedullary differentiation and hyperechoic thickening of the adipose tissue of the bilateral perirenal space. This pattern is generally observed in autosomal recessive polycystic kidney disease. The remaining abdominal organs studied through ultrasound did not present significant abnormalities.

Insulin-like growth factor 1 (IGF-1) was 267.1 ng/mL (normal range: 56.2-267.1 ng/mL), and its binding protein 3 (IGFBP3) was 3941 ng/mL (normal range: 1995-4904 ng/dL); these levels were not suggestive of growth hormone deficiency.

Renal function and glucose metabolism were normal. Liver function tests initially showed elevation of transaminases, with negative infectious and immunologic liver tests, which progressively improved until normalization at 6 years of age, although liver enlargement was present on ultrasonography, with diffuse irregular echostructure. These findings have not been described previously in patients with MOPDII and remain of uncertain origin.

**Figure 4:**
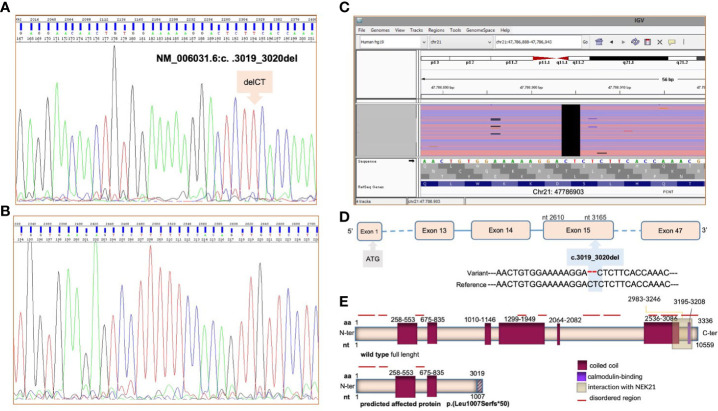
NGS and Sanger sequencing showed a novel homozygous c.3019_3020del deletion in the *PCNT* gene on genomic DNA. The forward DNA sequencing electropherogram of exon 15 and of the *PCNT* gene is reported in panel **(A)**, the reverse in **(B)**. Ref Seq (reference sequencing) was used for variant annotations: NM_006031.6 and NP_006022.3. The identified pathogenic variant in the *PCNT* gene, visualized using the Integrative Genome Viewer (IGV) software, is presented in **(C)**. In panels **(D, E)**, the identified mutation and the predicted consequence for the pericentrin protein are detailed. The pericentrin structure was derived from https://www.uniprot.org/uniprotkb/O95613/entry.

Following genetic counseling, genetic testing was carried out *via* next-generation sequencing on the PGM platform (Life Technologies), using an amplicon design covering all coding exons and exon–intron boundaries of the PCNT gene and following the DNA sample guidelines (https://varnomen.hgvs.org/). The DNA sample was obtained after the proband’s mother provided informed consent to the analysis. The molecular analysis identified the c.3019_3020del in exon 15 predicted to lead to a premature codon termination fifty bases after codon 1007, p.(Leu1007Serfs*50) and production of truncated protein (**Figures 4D, E**). This could not be verified experimentally due to the small amount of peripheral blood available from the proband. However, based on the American College of Medical Genetics and Genomics (ACMG) criteria (12), the identified variant can be classified as pathogenic because loss-of-function mutation in the PCNT gene is a known mechanism of disease (13) (PVS1). The ClinVar database classifies this variant as pathogenic (PP5), and homozygotes for this variant are absent in the GnomAD (PM2) database. The pathogenic variant identified by NGS technology was validated by Sanger sequencing (**Figures 4A–C**) and reported using the Human Genome Variation Society nomenclature guidelines (https://varnomen.hgvs.org/). This genetic variant has not been described previously in the literature.

**Figure 5:**
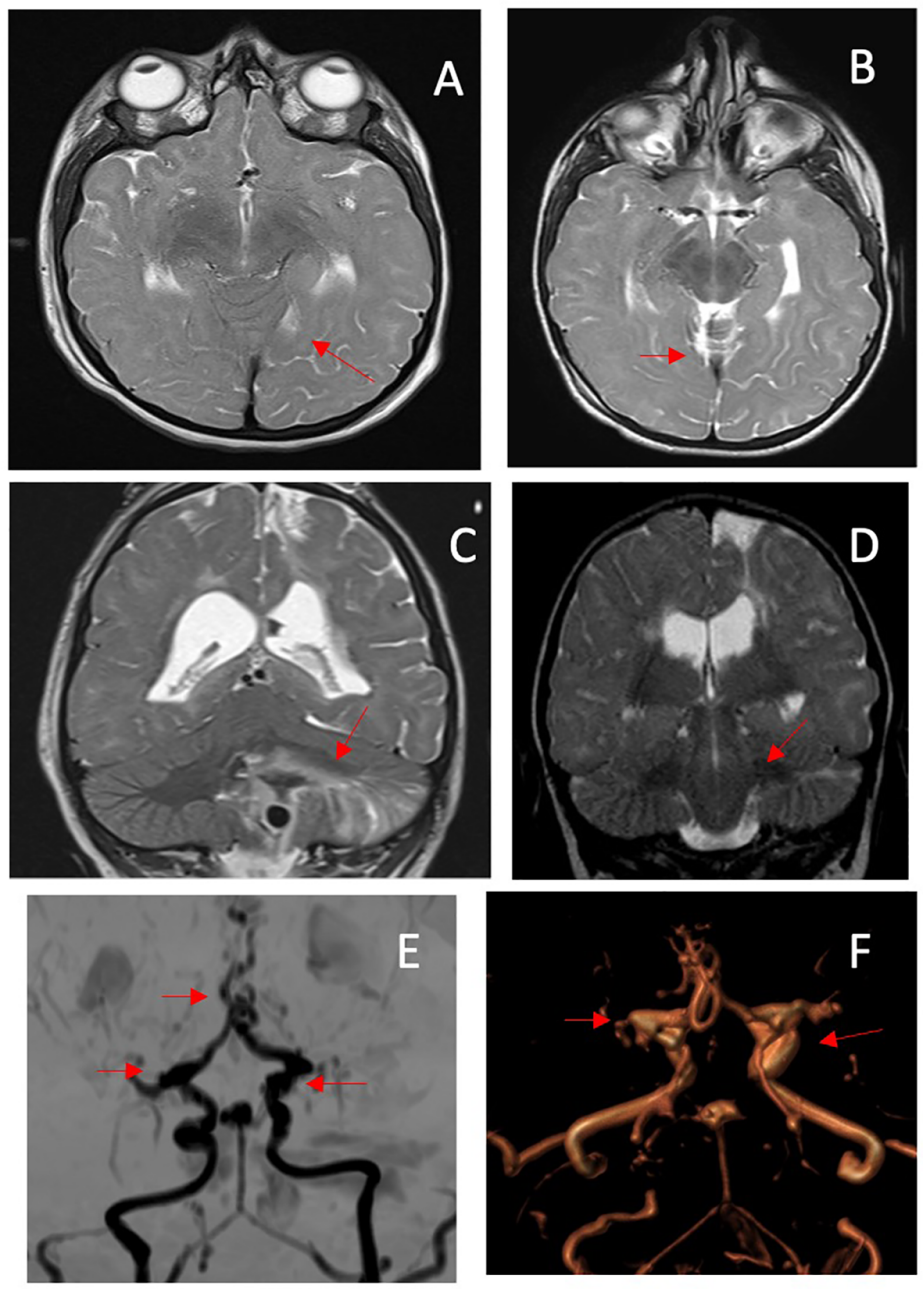
MRI performed at diagnosis. **(A)** Axial T2-weighted image (WI) showing a diffuse attenuation of the flow void of the middle cerebral arteries; **(B)** coronal T2WI shows multiple areas of prior infarction, seen as areas of high signal intensity. MRI performed at age 8 yr. **(C)** axial T2-WI shows a decrease in flow void of the anterior cerebral arteries; **(D)** an acute hemorrhagic infarction is also visible in the left cerebellar hemisphere on T2-WI coronal image. **(E, F)** MIP/3D TOF MRA: the coronal projection shows diffuse stenosis/dilatations of the vessels of the circle of Willis, with Moyamoya appearance, consisting in sub/occlusion of the middle cerebral arteries, stenosis of the anterior cerebral arteries (with a mouse-tail appearance), bilateral carotid siphons, basilar apex and tonsillar branch of the posterior-inferior cerebellar artery aneurysmal dilatation. The myriad of small collateral blood vessels including the rete mirabilis in the region of the perforating arteries is also visible. The red arrows point at the anomalies described.

Initial magnetic resonance imaging (MRI) of the brain was performed at diagnosis, and showed areas of gliosis with encephalomalacia in the frontal cortico-subcortical and left parasagittal parietal area; a diffused hypo/delayed myelination with non-specific signal alteration, in particular on the left cerebral hemispheric side; irregularity of the subependymal surface; a thinned corpus callosum at the level of the trunk; dilation of the lateral ventricles, especially at the level of the trigons; a thinned appearance of the middle and anterior cerebral arteries; and triangular and flattened shape of the frontal bones with trigonocephaly (**Figures 5A, B**).

Based on previous recommendations, as possible cerebral accidents were described at a later age (14), the child did not undergo further brain MRI scans until he presented at the age of 8 years with a brain hemorrhage due to the rupture of an aneurysm located in the posterior inferior cerebellar artery. The MRI conducted after the stroke showed the presence of multiple aneurysms located in the internal carotid, communicating arteries, and the apical portion of the basilar artery that had been absent during the first neuroimaging evaluation conducted when the child was 3 years old. The MRI also highlighted an occlusion of the M1 segment of both middle cerebral arteries and an irregular and stenotic appearance of the A3-A4 segments of the anterior cerebral arteries associated with the development of a collateral vessel network (Moyamoya disease) (**Figures 5C–F**). Unfortunately, the child died as a result of this acute event.

## Discussion

MOPDII is the most common form of microcephalic primordial dwarfism (MPD) and differs from other similar diseases, such as Seckel syndrome, in that it is characterized by more severe growth impairment, skeletal dysplasia, and less severe intellectual disability. *PCNT* gene variants are causative for this condition. Pericentrin is a multifunctional protein that plays both a structural role, as a major constituent of pericentriolar material, and a functional role in recruiting other proteins for cell cycle progression. Indeed, *PCNT* participates in the formation of the mitotic spindle as a core centrosomal component, but also regulates cellular proliferation through interactions with other signaling pathways that control cell cycle checkpoints and mitotic entry. Recent studies conducted in patients with MOPDII have additionally shown defective ataxia–telangiectasia and a Rad3-related protein (ATR)-dependent DNA damage response, as a consequence of an ectopic localization of checkpoint kinase 1 from the centrosome due to *PCNT* gene mutations. ATR was the first mutated gene discovered in patients with MPD: it encodes a protein kinase (PI3K) that regulates the DNA damage response pathway, and it is now also associated with Seckel syndrome (10).

Despite the heterogeneous molecular basis of these conditions, MOPDII shares some features with other disorders belonging to the MPD group (Seckel syndrome, MOPD I/III, and Meier-Gorlin syndrome), such as severe pre- and post-natal growth retardation and marked microcephaly, in addition to the characteristic facies, skeletal dysplasia, abnormal dentition, diabetes/insulin resistance, and increased risk for neurovascular disease. In the case we describe, no metabolic issues were identified, possibly because of the patient’s young age, but a close endocrinological follow-up was scheduled in order to identify these possible complications early.

The first brain MRI in our patient highlighted a thinned appearance of the middle and anterior cerebral arteries. Central nervous system vascular anomalies have been reported as an important cause of morbidity and mortality in patients with MOPDII (7), and Moyamoya disease has also been reported during childhood; however, aneurysmal disease has been described frequently, with a mean age of appearance of approximately 9.3 years (1, 15). Overall, nervous system vascular anomalies are reported to be an important cause of morbidity and mortality in patients with MOPDII (1).

This patient, however, died of a brain hemorrhage at the age of eight, highlighting the need for more careful and closer neuroimaging follow-up, which needs to be completed *via* specific angiographic studies.

The most recent review, published in May 2021 and covering 47 American patients, reported that 47% of these subjects were diagnosed with Moyamoya or intracranial aneurysms at a mean age of 9.3 years, and 19 underwent Moyamoya intravascular bypass surgery; 53% had aneurysms identified, but 36% had none of these vascular anomalies (9). The authors of this review recommended brain MRI evaluations early in childhood (16) (in particular, once yearly during the first decade of life), and thereafter according to previous findings, without exceeding 18-month intervals between investigations, for early identification of vessel anomalies in order to allow the use of both surgical and pharmacological approaches (anticoagulants) (9). More recent publications suggest that brain MRI should be carried out at the moment of diagnosis (1). This case report confirms the need for such close follow-up of brain MRI.

In this patient, kidney abnormalities were also identified on ultrasound. The findings were similar to those of autosomal recessive polycystic kidney disease. Renal involvement is not usually described in MOPDII, although rare cases of nephrolithiasis have been reported (15), and a very recent publication by Hettiarachchi et al. has described a child with a novel distinct mutation in the pericentrin gene associated with bilaterally small kidneys, without other renal anomalies (17).

It can be hypothesized that severe variants in the *PCNT* gene could lead to centrosome disruption, including mislocalization of the centrosome protein and proteins involved in cilia genesis, potentially contributing to cyst formation. Hall et al. reported observing unilateral cystic dysplasia of the kidney in a male patient with MOPDII, although the *PCNT* gene mutations were unknown at that time in that patient (18). Renal involvement with autosomal recessive polycystic kidney disease pattern, as observed in our patient, has never been described in children with MOPDII. However, further reports and studies are warranted to clarify this aspect, and to provide further understanding of liver function.

## Conclusions

This case report identifies a novel *PCNT* gene mutation associated with MOPDII that is predicted to cause severe derangement in protein structure and function, explaining the severe features of the condition observed in the patient, who (in addition to characteristics described in previous reports on this syndrome) presented with early severe neurovascular disease, kidney abnormalities, and possible transient/relapsing changes in liver function. Considering the impact of neurovascular disease on morbidity and mortality in these patients, close follow-up *via* brain MRI associated with angiography is mandatory for early identification of vessel anomalies and determination of appropriate management.

## Data availability statement

The original contributions presented in the study are included in the article/supplementary materials. Further inquiries can be directed to the corresponding author.

## Ethics statement

Written informed consent was obtained from the minor(s)’ legal guardian/next of kin for the publication of any potentially identifiable images or data included in this article.

## Author contributions

MPe wrote the first draft of the manuscript and was in charge of the patient’s follow-up. EC, MG, GM and LB contributed to clinical activities and performed the literature analysis. VDP made scientific contributions. MPi and AP performed the genetic analysis and FO prepared, evaluated, and annotated the neuroimages. SMRE supervised the outpatient clinic activities. MS critically revised the text and made substantial scientific contributions. All authors contributed to the article and approved the submitted version.

## References

[1] Duker A Jackson A Bober M. Microcephalic osteodysplastic primordial dwarfism type II. In: Adam MP, editors. GeneReviews. University of Washington, Seattle (2021). 34978779

[2] Hall JG Flora C Scott CI Jr Pauli RM Tanaka KI. Majewski osteodysplastic primordial dwarfism type II **(**MOPD II): natural history and clinical findings. Am J Med Genet A (2004) 130A(1):55–72. doi: 10.1002/ajmg.a.30203 15368497

[3] Karatas AF Bober MB Rogers K Duker AL Ditro CP Mackenzie WG. Hip pathology in majewski osteodysplastic primordial dwarfism type II. J Pediatr Orthop (2014) 34(6):585–90. doi: 10.1097/BPO.0000000000000183 24705347

[4] Rigter LS El Moumni M Ten Duis HJ Wendt KW. Fracture healing of an osteodysplastic femur in a microcephalic osteodysplastic primordial dwarfism II (MOPD II) patient: a case report. Eur J Pediatr Surg (2013) 23(4):327–9. doi: 10.1055/s-0032-1315810 22773355

[5] Duker AL Kinderman D Jordan C Niiler T Baker-Smith CM Thompson L. Microcephalic osteodysplastic primordial dwarfism type II is associated with global vascular disease. Orphanet J Rare Dis (2021) 16(1):231. doi: 10.1186/s13023-021-01852-y 34016138 PMC8139163

[6] Brancati F Castori M Mingarelli R Dallapiccola B. Majewski osteodysplastic primordial dwarfism type II **(**MOPD II) complicated by stroke: clinical report and review of cerebral vascular anomalies. Am J Med Genet A (2005) 139A:212–5. doi: 10.1002/ajmg.a.31009 16278902

[7] Bober MB Jackson AP. Microcephalic osteodysplastic primordial dwarfism, type II: a clinical review. Curr Osteoporos Rep (2017) 15(2):61–9. doi: 10.1007/s11914-017-0348-1 PMC5561166 28409412

[8] Piane M Della Monica M Piatelli G Lulli P Lonardo F Chessa L. Majewski osteodysplastic primordial dwarfism type II **(**MOPD II) syndrome previously diagnosed as seckel syndrome: report of a novel mutation of the PCNT gene. Am J Med Genet A (2009) 149A(11):2452–6. doi: 10.1002/ajmg.a.33035 19839044

[9] Willems M Geneviève D Borck G Baumann C Baujat G Bieth E. Molecular analysis of pericentrin gene (PCNT) in a series of 24 seckel/microcephalic osteodysplastic primordial dwarfism type II (MOPD II) families. J Med Genet (2010) 47(12):797–802. doi: 10.1136/jmg.2009.067298 19643772

[10] Delaval B Doxsey SJ. Pericentrin in cellular function and disease. J Cell Biol (2010) 188(2):181–90. doi: 10.1083/jcb.200908114 PMC2812529 19951897

[11] Varsome . Available at: https://varsome.com/gene/hg19/pcnt (Accessed 23.01.2023).

[12] Richards S Aziz N Bale S Bick D Das S Gastier-Foster J. Standards and guidelines for the interpretation of sequence variants: a joint consensus recommendation of the American college of medical genetics and genomics and the association for molecular pathology. Genet Med (2015) 17:405–24. doi: 10.1038/gim.2015.30 PMC4544753 25741868

[13] Rauch A Thiel CT Schindler D Wick U Crow YJ Ekici AB. Mutations in the pericentrin (PCNT) gene cause primordial dwarfism. Science (2008) 319:816–9. doi: 10.1126/science.1151174 18174396

[14] Perry LD Robertson F Ganesan V. Screening for cerebrovascular disease in microcephalic osteodysplastic primordial dwarfism type II (MOPD II): an evidence-based proposal. Pediatr Neurol (2013) 48(4):294–8. doi: 10.1016/j.pediatrneurol.2012.12.010 23498563

[15] Bober MB Khan N Kaplan J Lewis K Feinstein JA Scott CI Jr.. Majewski osteodysplastic primordial dwarfism type II (MOPD II): expanding the vascular phenotype. Am J Med Genet A (2010) 152A(4):960–5. doi: 10.1002/ajmg.a.33252 20358609

[16] Hildebrandt F Otto E. Cilia, and centrosomes: a unifying pathogenic concept for cystic kidney disease? nat. Rev Genet (2005) 6:928–40. doi: 10.1038/nrg1727 16341073

[17] Hettiarachchi D. Subasinghe S. M. V. Anandagoda G. G. Panchal Hetalkumar Lai P. S. Dissanayake V. H. W. Novel frameshift variant in the PCNT gene associated with microcephalic osteodysplastic primordial dwarfism (MOPD) type II and small kidneys. BMC Med Genomics (2022) 15:182. doi: 10.1186/s12920-022-01226-8 35422036 PMC9009051

[18] Hall JG Flora C Scott CI Jr Pauli RM Tanaka KI. Majewski osteodysplastic primordial dwarfism type II (MOPD II): natural history and clinical findings. Am J Med Genet A. (2004) 130A(1):55–72. doi: 10.1002/ajmg.a.30203 15368497

